# Is the Race Decaying?

**Published:** 1913-01-11

**Authors:** 


### IS THE RACE DECAYING?

"There were giants in the earth in those days." So says the Bible, placing the days of the giants in some prehistoric past. So, in effect, has the elder generation always said when talking to the younger. We are not, we are told, as tall or as strong as our forefathers, we do not eat as heartily, nor drink as deep, and we could not, if we were put to it, fight as well. We are not prolific as of yore, though Havelock Ellis has done much to counter the scare on this head.

In this there is much of the mere talk of the grumbler, and some of the pathetic illusion which ever surrounds our youthful days as we grow old; but it must be admitted that there is in it all a substratum of truth. The Anglo-Saxon race, not only in England, but in our oversea possessions and in America, is far less prolific than formerly. Fewer children are born, and though more of those who are born survive, the survivals do not wholly make up for the unborn. Fecundity is not a matter on which personal and national prosperity walk side by side. The point of view of the average parent is this: "If I have only two or three children I can give them a good education, and, perhaps, leave them enough to keep them from want, while I could not give a large family a fair start in life." From the national point of view the matter may be stated thus: "A race which tries to do no more than make up for the places that are emptied by death is likely in the end to fail to do even that; it is making for the vanishing point, and the very comfort which parents desire to give their children makes them so much the less hardy, and less able to withstand unavoidable ills." One would fain avoid wastage in humanity, but some allowance for inevitable wastage must be made, and civilised nations as a rule are failing to consider the point.

### The Infant Mortality Question.

It may indeed be argued with reason that we do not need to allow so much for this as of old. Our infant mortality is still far too great, but it is much less than it was a hundred years ago. We do manage to rear a fair proportion of the children who are born, and, therefore, a declining birth-rate need not necessarily be alarming.

Nevertheless, there is cause for anxious thought in the fact that the birth-rate is decreasing, especially among the middle classes, who have so long and so loudly asserted that they are the backbone of the nation. The richer classes may indulge in as many children as they like, because they have enough to educate and endow them. And the poorer not only leave education and all other forms of provision to the State or charity, but look upon a large family as an insurance fund—providing workers to support them in old age. But the man of the middle class, who is not endowed by fortune, who "cannot dig, and to beg is ashamed," whose whole wealth is in his brain and the use he can make of it, declines in these days to complicate the problem by a numerous progeny. It is a pity, for the "backbone" boast is not wholly unfounded, and it is in the middle classes that we most frequently find the combination of intellect and energy, a combination due in all probability to the union of suitable heredity with strong necessity.

Divers reasons, selfish and social, combine to make the men and women of this class late in marrying, and if man or woman marries at thirty instead of twenty their families are likely to be smaller. The mere reduction of procreative life by ten years need not in itself be harmful. In old days observers were wont to note that the younger children of a family were cleverer and more vigorous than their seniors, and a schoolmaster of twenty years' standing told the present writer that in his experience younger sons were, as a rule, brighter than their elder brothers. This would imply that the best children were born when their parents had reached full maturity. If the age of the parents alone were the determining feature of a child's quality we might safely hope that the first child of a mother of thirty and a father of thirty-five would be as good as the second, third, or fourth of their parents. But, unfortunately, we cannot so easily size up the matter. In the first place, the muscles of the primipara of thirty are stiffer than those of a multipara of the same age and instrumental interference—never a good thing, though often necessary—may be required at the birth. An injury may thus be done to the child which does not appear at the moment, but may affect future development, mental or physical. An even more serious injury may be done to the children of these mature marriages by the admitted and practically permitted pre-marital self-indulgence of the father. Even when the organism has not been actively infected as a result of indulgence, there are too many men in whom the sequelæ of venereal disease are present, even when the disease itself has been cured, and affect injuriously both wife and children.

The Postponement of Marriage.

We can hardly claim that the postponement of marriage is a physiological success, even though it may lead to material prosperity. The philosophers who recommended late marriages as a means of restricting population always assumed that both sexes would live chastely before marriage—an assumption which is not borne out by the facts of life.

Another remedy, and one that is increasingly popular, is not to postpone marriage, but only the bringing of children into the world. Sometimes a child comes in a reasonable time, and then the parents, realising the cost of up-bringing, determine that there shall be no more. Sometimes it is resolved to postpone the advent of the child until the income increases. This idea has developed a custom which our fathers and mothers would have probably described in a general way as "French vices," the use of drugs or other means to prevent conception. Perhaps only gynæcologists could tell us what the result of this habit has been, and they can only speak in general terms. But one cannot but know that much of the so-called "nervous prostration" of women is thought by some to be due to undue nervous excitation, made safe, as it is thought, by the means we have alluded to. It is, however, an argument incapable of proof, like the *post hoc ergo propter hoc* allegation of those who attribute appendicitis to eating steel-ground flour, boric acid preservatives, tinned meats and sweets. It is the over-excitement of extreme indulgence and the starvation of natural instincts which chaste spinsterhood may incur that alike produce neurosis. Unfortunately excessive indulgence has no monopoly.

Meanwhile, the lazy, the improvident, the unfit go on propagating their kind unchecked, secure that modern philanthropy will prevent their suffering to the full those dire effects of starvation, insanitary dwellings, and general neglect that offered a check to their multiplication in days gone by. We have not yet got to the point of preventing the permanently weak-minded, the consumptive, the patient who has only intervals of sanity from begetting his like. Meanwhile, the temperate, the thoughtful, the provident say that they "cannot afford" to have children. It has occurred to historians that the history of the Middle Ages might have been less sanguinary and more quickly progressive if the temperate and refined members of it had not felt impelled to take refuge in monasteries and convents from the rudeness and cruelty of their time. But the same people are now, without the excuse of a rude and cruel age, shirking the duty of giving children of their own kind to their nation. With us the sin is, if possible, more inexcusable than with other nations. We believe in our mission to the world—that it is our particular business to occupy the waste places of the earth, and to give the benefits of civilisation and good government to inferior races. Do we not continually declare our conviction that this is the case? But do we try to fulfil our mission? Our Colonies cry out for emigrants, and we send them the "remittance man"—the man who has disgraced himself and his family, and is sent abroad with a small income—at the one end of the social scale, and the weedings of the slums at another. And then we grumble at the prolific Russians, Germans, Poles, and Italians invading our Colonies in their thousands, and filling up the waste places which we call our own.

Where is the Remedy?

To be frank, the remedy lies in the decrease of luxury, combined with the more ready acceptance of such advantages as the State can give. Continental countries have already reached the latter point. It is no disgrace in Germany to be educated at a State school, and the small fees charged at the Real-Schule bear no relation to the cost of the education. But where is the English mother of the upper-middle class who would consent to her son sitting side by side with the sons of the tradesmen who supply the daily needs of her household? She will rather pay fees which she cannot afford for inferior education than let her child go to the Board or Council school. A servant or two, although loudly declared to be more of a trial than a comfort, are necessary to her social position. Both husband and wife regard as necessaries things which to their parents would have seemed luxurious in the extreme, and both excuse themselves on the plea that "times have changed." Is it true that England has lost her *bourgeoisie*, her people who fulfilled the duties of their sphere without discontent with their own station or ambition after the appearances of another? Women have been largely to blame for the change. For the sake of the appearances of life they have starved themselves and all belonging to them of the realities. If the "woman movement" of to-day, which professes to glorify motherhood, will really induce its votaries to throw off the slavery of custom, and return to the primitive and natural duties and responsibilities of family life, it will, vote or no vote, have done good to the nation.

As regards a decreased demand for luxuries, there is little hope that this will ever occur. Indeed, since the great man is the man who creates a new need—steam travel, penny posts, the telephone, a national theatre are all examples from here or abroad—it would not be right to lament this uncritically. Civilisation is the creation of new needs. How can it be maintained when it is the least efficient to survive who procreate without any check, save the high mortality that usually accompanies a reckless birth-rate?

